# Stereotactic radiosurgery for brain metastases: analysis of outcome and risk of brain radionecrosis

**DOI:** 10.1186/1748-717X-6-48

**Published:** 2011-05-15

**Authors:** Giuseppe Minniti, Enrico Clarke, Gaetano Lanzetta, Mattia Falchetto Osti, Guido Trasimeni, Alessandro Bozzao, Andrea Romano, Riccardo Maurizi Enrici

**Affiliations:** 1 Department of Radiation Oncology, Sant' Andrea Hospital, University "La Sapienza", Rome, Italy; 2 Department of Neurological Sciences, Neuromed Institute, Pozzilli (IS), Italy; 3 Department of Neuroradiology, Sant' Andrea Hospital, University "La Sapienza", Rome, Italy

**Keywords:** brain metastases, stereotactic radiosurgery, survival, radiation-induced complications, brain necrosis

## Abstract

**Purpose:**

to investigate the factors affecting survival and toxicity in patients treated with stereotactic radiosurgery (SRS), with special attention to volumes of brain receiving a specific dose (V10 - V16 Gy) as predictors for brain radionecrosis.

**Patients and Methods:**

Two hundred six consecutive patients with 310 cerebral metastases less than 3.5 cm were treated with SRS as primary treatment and followed prospectively at University of Rome La Sapienza Sant'Andrea Hospital. Overall survival, brain control, and local control were estimated using the Kaplan-Meier method calculated from the time of SRS. Univariate and multivariate analysis using a Cox proportional hazards regression model were performed to determine the predictive value of prognostic factors for treatment outcome and SRS-related complications.

**Results:**

Median overall survival and brain control were 14.1 months and 10 months, respectively. The 1-year and 2-year survival rates were 58% and 24%, and respective brain control were 43% and 22%. Sixteen patients recurred locally after SRS, with 1-year and 2-year local control rates of 92% and 84%, respectively. On multivariate analysis, stable extracranial disease and KPS >70 were associated with the most significant survival benefit. Neurological complications were recorded in 27 (13%) patients. Severe neurological complications (RTOG Grade 3 and 4) occurred in 5.8% of patients. Brain radionecrosis occurred in 24% of treated lesions, being symptomatic in 10% and asymptomatic in 14%. On multivariate analysis, V10 through V16 Gy were independent risk factors for radionecrosis, with V10 Gy and V12 Gy being the most predictive (p = 0.0001). For V10 Gy >12.6 cm^3 ^and V12 Gy >10.9 cm^3 ^the risk of radionecrosis was 47%.

**Conclusions:**

SRS alone represents a feasible option as initial treatment for patients with brain metastases, however a significant subset of patients may develop neurological complications. Lesions with V12 Gy >8.5 cm^3 ^carries a risk of radionecrosis >10% and should be considered for hypofractionated stereotactic radiotherapy especially when located in/near eloquent areas.

## Introduction

Stereotactic radiosurgery (SRS) has become an increasingly treatment option in the initial management of patients with brain metastases. Its efficacy when used alone or in combination with whole brain radiation-therapy (WBRT) has been demonstrated in several randomized trials and multi-institutional studies [1-8]. SRS plus WBRT is associated with better local tumor control and functional autonomy for patients with brain metastases when compared to WBRT alone, and with significant longer survival in patients with a single metastasis [3]. Recently, two large randomized studies have shown similar survival benefits and functional independence between patients with 1-3 brain metastases treated with SRS alone and SRS plus WBRT [7,8].

The reported survival of 7-14 months after SRS is roughly equivalent to that reported after surgical resection [9]. Although surgery is usually indicated in patients with lesions causing significant mass effect and for large lesions > 3 cm in locations amenable to resection, in current clinical practice SRS is frequently employed as less invasive and more cost-effective treatment option than resection.

A variable rate of neurological complications of 2-14% has been reported after SRS [7,8,10-17]; however, a higher rate has been shown in some studies [1,18-20] suggesting that patients may have side-effects after SRS more often than reported. The most common complication of SRS is represented by the development of brain radionecrosis that may occur in up to 50% of treated lesions [21-26]. Factors related to the development of radionecrosis after SRS include dose, treated volume, and volume of the brain receiving a specific dose [22,23,25-28]

In the present study we have reviewed our experience with SRS in patients with brain metastases treated with SRS alone as primary treatment. Related factors associated with the clinical outcome and the development of treatment-induced complications have been evaluated.

### Patients and Methods

Between September 2006 and January 2010, 206 consecutive patients aged 18 years or older with 1-3 cerebral metastases less than 3.5 cm on contrast-enhanced magnetic resonance imaging (MRI), and derived from an histologically confirmed systemic cancer, were treated with SRS as primary treatment and followed prospectively at University of Rome La Sapienza Sant'Andrea Hospital. Patients who had received previous surgical resection or WBRT, or receiving adjuvant WBRT following SRS were excluded from the study.

All metastatic tumors were treated with LINAC-based SRS. The BrainLAB frameless stereotactic system, in conjunction with the BrainScan treatment planning system (Version 5.31) has been used for stereotactic treatment. The target volume was identified on the basis of the fused CT and magnetic resonance image (MRI) scans. Radiosurgical dose was 20 Gy for metastases with a volume ≤ 4.3 cm^3 ^(corresponding to a sphere of 2 cm in diameter), 18 Gy for metastases with a volume of 4.3-14.1 cm^3^, and 15-16 Gy for metastases with a volume > 14.1 cm^3 ^or located in the brainstem. The gross tumor volume (GTV) was delineated as a contrast-enhancing tumor demonstrated on MRI scans. The planning target volume (PTV) was generated by the geometric expansion of GTV plus 1-2 mm. Doses were prescribed to the 80-90% isodose line normalized to the maximum dose. Treatment volumes were achieved with 6-10 noncoplanar dynamic arcs by using a 6-MV LINAC. All patients underwent a second CT (verification CT) scan before the start of treatment in the CT-room and immediately transferred to the treatment room in a wheel chair. Planning and verification CT scans were fused employing a fusion algorithm included in the BrainLab planning system. The new coordinates of the isocenter were recorded and the isocenter shift between verification and planning CT calculated as previously reported [29]. This whole procedure takes less than 10 minutes. The mask was refitted or the treatment replanned if the isocenter shift was > 1.0 mm. Patients with multiple metastases were treated in 2 or 3 following days in outpatient clinic.

Patients were examined clinically one month after radiosurgical treatment and then every 2 months. MRI was made every 2 months in the first year after the treatment, and then every 3 months or as appropriate according to the neurological conditions. The size of treated lesions was measured in three dimensions. Complete and partial responses were defined as total radiographic disappearance of lesion or decrease in tumor volume > 50%. Local progression was defined as radiographic increase in the size of metastatic lesion. For all patients who died, the cause of death (intracranial versus extracranial progression) was determined by clinical/neurological evaluation and brain/systemic radiologic studies. Patients were considered to have died as result of a neurological death if they had evidence of progressive intracranial disease consisting of expanding intracranial masses, CNS hemorrhage, progressive neurologic symptoms, meningeal carcinomatosis, or hydrocephalus resulting in herniation.

At each visit, neurological status and the severity of complications were rated according to RTOG CNS toxicity criteria. Severe complications were considered to have an RTOG Grade ≥ 3). Adverse neurological events were considered consequence of SRS treatment if they were associated or not to radiological abnormalities suggestive of brain radionecrosis in absence of progressive disease. Radionecrosis was assessed subjectively using anatomic and dynamic susceptibility-weighted contrast-enhanced (DSC) perfusion MRI. The following criteria have been considered as suggestive of radionecrosis: 1) increased T1 contrast enhancement located in the irradiated area with central hypointensity and increased peripheral edema; 2) substantial regression or stability (for at least 4 months) of enhancing areas on serial follow-up MRI scans without additional treatment; 3) a clear absence of perfusion (black hole), in the absence of any nodular highly vascularized area within the contrast-enhanced lesion at perfusion MRI. Enhancing lesion that progressively increased in size on serial MR imaging during a minimum follow-up period of 4 months was scored as recurrent metastatic tumor. All diagnoses were confirmed retrospectively by the same experienced neuroradiological team (AB, AR, GT). Radionecrosis was recorded as clinically symptomatic when associated with neurological deterioration, whereas was recorded as asymptomatic in patients who remained neurologically stable.

### MRI protocol

All MRI scans were obtained with a 1.5-T MRI scanner (Siemens Sonata, Siemens Medical Systems, Erlangen, Germany). After a localizing sagittal T1-weighted image, non-enhanced axial T1-weighted spin echo (TR/TE, 600/12 ms) and axial T2-weighted (TR/TE 3,680/85) images were obtained. Post-contrast axial and sagittal (multiplanar reconstruction) T1-weighted imaging was performed after the acquisition of the DSC MRI data. DSC MRI scans were acquired using a T2-weighted (TR/TE/flip angle:1.490/40/90°) EPI sequence. A dynamic image series of 50 measurements performed on 14 axial sections with slice thickness 5 mm and interslice gap 1.5 mm resulted in a total scan time of 1.20 min, with a field of view of 230-230 mm, matrix 128-128 and an image acquisition matrix of 128 × 128, signal bandwidth 1502. A dose 0.1 mmol/kg bolus injection of gadolinium contrast (Magnevist; Shering Diagnostics, Berlin, Germany) delivered at the rate of 5 ml/s was used. The post-processing of the DSC MRI data were performed on a Leonardo VD10B Syngo OEM installation (Siemens AG).

### Data analysis

Overall survival, brain control, and local control (control of irradiated lesions) were estimated using the Kaplan-Meier method calculated from the time of SRS. For univariate analysis, the log-rank test was used for categorical variables, and the Cox proportional hazards model was used for continuous variables. The following factors for outcome were tested: age (<65 *vs *≥65 years), pretreatment KPS score (≤70 *vs *>70), number of brain metastases (1 *vs *> 1), recursive partitioning analysis (RPA) class (I *vs *II *vs *III), histology (lung *vs *breast vs melanoma *vs *others), and extracranial disease (stable *vs *active). Radionecrosis changes were assessed per tumor and event-free survival time using the Kaplan-Meier method. Univariate analysis was performed to identify risk factors for the presence of radionecrosis by using the following patient and tumor determinants: sex, age, histology, KPS score, tumor volume, SRS dose, volume receiving a specific dose of 10,12,14,16 and 18 Gy (V10 Gy-V18 Gy), site of tumor, conformality index [30], and homogeneity index. Prognostic factors for treatment outcome and SRS-related complications found significant (P < 0.05) were included in a multivariate outcome with analysis performed using a Cox proportional hazards regression model. In order to compare the own results with previously published risk prediction models, we have analyzed the correlation between V10 and V12 Gy and the increased risk of brain necrosis. Volumes were divided in intervals determined by quantiles and the risk of necrosis calculated in each interval. A probability value < 0.05 was considered statistically significant.

## Results

### Patients and tumor characteristics

Two hundred six patients (109 males and 97 females) with 310 metastases who underwent SRS between September 2006 and January 2010 and who met the previously described inclusion criteria were analyzed. Tumor characteristics are listed in Table 1. One hundred twenty-six patients (61%) were treated for 1 metastasis, 56 (27%) for 2 metastases, and 24 patients (12%) for 3 metastases. The median age at the time of SRS was 62 years (range 26-81). The most common histologies were lung, breast, and melanomas. The most common location was parietal lobe followed by frontal and temporal lobe. According to RTOG recursive partitioning analysis (RPA) classes for brain metastases, 49 (24%) patients were in RPA Class I, 133 (65%) patients in RPA Class II, and 24 (11%) patients in RPA Class III. One hundred and fifty-six patients received chemotherapy before treatment or during the subsequent follow-up. Data were reported to September 2010. At this time 91 patients were alive.

**Table 1:** Summary of tumor characteristics and treatment parameters

Parameter	No(%)
***number of patients***	206
***median age***	62
***sex (F/M)***	99/107
***no of lesions per patient***	
1 lesion	126 (61%)
2 lesions	56 (27%)
3 lesions	24 (12%)
***histology***	
lung	106 (51%)
breast	38 (18%)
melanoma	34 (17%)
others	28 (14%)
***tumor location***	
frontal	68 (22%)
parietal	78 (25%)
temporal	62 (20%)
cerebellar	43 (14%)
occipital	45 (15%)
brainstem	14 (4%)
***radiosurgical dose (Gy)***	
20	118 (38%)
18	120 39%)
15-16	72 (23%)
***treated volume (cm***^***3***^***)***	
median	1.88
range	0.03-18.1
***treated volume (cm***^***3***^***)***	
median	2.81
range	0.2-23.7

The median GTV was 1.88 cm^3 ^(range 0.03-18.1 cm^3^), and the median PTV was 2.81 cm^3^(range 0.2-23.7 cm^3^). Mean prescribed dose was 18 Gy (range 15-20 Gy) at a median isodose of 87% (range 84-91). The average homogeneity index was 1.1 (range 1-1.3), and the median conformality index was 1.6 (range 1.1-2.7).

### Overall survival and brain control

**Figure 1:**
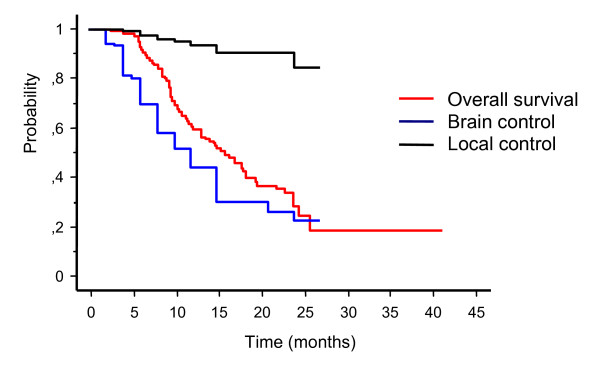
Kaplan-Meier analysis of overall survival, brain control, and local control

At a median clinical follow-up of 9.4 months (range 2-42 months) median survival and brain control were 14.1 months and 10 months, respectively (Figure 1). The 1-year and 2-year survival rates were 58% and 24%, and respective brain control rates were 43% and 22%. Seventy-nine percent of patients succumbed to their extracranial disease and 21% of patients died of progressive intracranial disease. Intracranial tumor progression at either distant or local sites in the brain was observed in 74 patients. Sixty-three patients had new brain metastases at distant sites. The 6-month and 12-month actuarial rates of developing new brain metastases were 26% and 50%, respectively. Sixteen patients recurred locally after SRS. The 1-year and 2-year local control rates were 92% and 84%, respectively. Salvage WBRT was applied in 47 patients and salvage SRS in 21 patients. Ninety-two (30%) metastases had a complete response, 106 (34%) had a partial response, and 112 (36%) remained stable. A clinical neurological improvement of pre-RT existing symptoms was recorded in 26 out of 77 patients (34%) during the follow-up.

Analysis of prognostic factors showed that extracranial disease, KPS, number of metastases, and RPA class were significant predictive factors for survival (Table 2). Histopathological type, age, and sex were not shown to be a significant factor. On multivariate analysis stable extracranial disease and KPS > 70 were associated with the most significant survival benefit. RPA class was not included in the multivariate analysis because it is not independent of age, KPS and extracranial disease status. Univariate analysis showed that control of extracranial disease (P = 0.01), KPS > 70 (P = 0.03), and number of metastases (1 vs >1, P = 0.01) were significant predictive factors for brain control; however, only extracranial disease (P = 0.001) and number of metastases (P = 0.03) were independent predictors on multivariate analysis. No significant prognostic factors were associated with local control.

**Table 2:** Univariate and multivariate survival analysis

Variable	No. of patients	Survival time Median months	univariate analysis P value	Multivariate analysis	
				Hazard ratio (95% CI)	P value
**Sex**			0.1		
Male	109	13.5			
Female	97	14.7			
**Age (years)**			0.1		
< 65	122	14.6			
≥ 65	84	13.3			
**KPS**			<0.0001	2.2 (1.4-3.4)	0.007
≤ 70	95	10.1			
>70	111	16.1			
**No of brain metastases**			0.03	1.5 (1-2.2)	0.1
1	126	14.7			
2-3	80	13.1			
**Primary tumor**			0.2		
Lung cancer	106	13.9			
Breast cancer	38	18.2			
Melanoma	34	12.7			
Others	28	13.1			
**Extracranial disease**			<0.0001	3.1 (1.8-5.0)	<0.001
stable	90	17.2			
active	116	9.8			
**RPA Class**			<0.0001		
Class I	49	19.2			
Class II	133	11.3			
Class III	24	7.3			

### Analysis of complications

Brain radionecrosis, as suggested by MR imaging or confirmed by histology (n = 12), was the most important complication occurring in 75 (24%) out of 310 treated lesions. Radionecrosis was symptomatic in 31 (10%) and asymptomatic in 44 (14%) of the treated lesions. Median time to symptomatic and asymptomatic necrosis were 11 months (range 2-32 months) and 10 months (range 2-30 months), respectively. Neurological deficits associated with radionecrosis including seizure, motor deficits, cognitive deficits, and speech deficits are shown in Table 3. Seizures occurred in 3 patients without evidence of any radiological change suggestive of radionecrosis. Overall, neurological complications were recorded in 28 (13.5%) patients, being severe (RTOG Grade 3 and 4) in 12 (5.8%) patients and requiring surgery or medical treatment. Steroid dependency occurred in 34 patients, with 16 patients who received high-dose dexamethasone for more than 4 months. Other complications were represented by headache, hydrocephalus, hemorrhage in 5%, 2%, and 2%, respectively. Overall, neurological and nonneurological complications occurred in 23% of patients.

**Table 3:** Incidence of complications associated with SRS among 310 metastases

Type of complication*	No/Total (%)
seizure	16 (5.2)
motor deficits	9 (2.9)
sensor deficits	4 (1.3)
cognitive deficits	3 (1.0)
speech deficits	4 (1.3)
visual deficits	2 (0.6)
ataxia	5 (1.6)
headache	15 (5)
nausea	3 (1.0)
hemorrhage	5 (1.6)
Cushing syndrome	7 (2.3)

* Twelve patients had multiple neurological deficits

**Figure 2:**
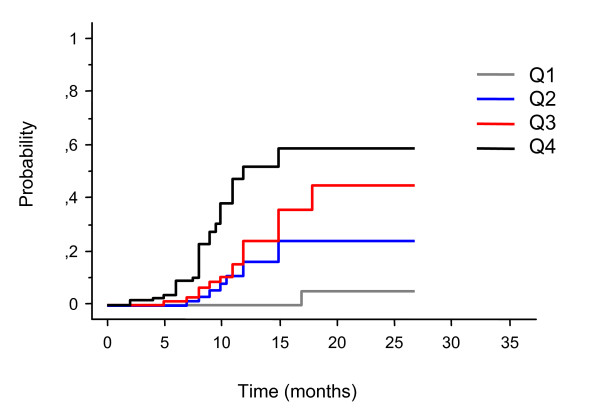
**Risk of brain radionecrosis after stereotactic radiosurgery for brain metastases in relation to brain volumes receiving 12 Gy (V12 Gy) stratified for quartiles (Q1-Q4)**. The risk increased significantly through Q1-Q4, corresponding to V12 Gy < 3.3 cm^3^, 3.3-5.9 cm^3^, 6.0-10.9 cm^3^, and >10.9 cm^3^, respectively. The actuarial risk at 1 year was 0% for Q1, 16% for Q2, 24% for Q3, and 51% for Q4

Univariate analysis showed that KPS, tumor volume, parietal location, and V10 through V16 Gy were significant variables for either symptomatic or asymptomatic brain necrosis (Table 4). The results of the Cox regression analysis showed that V10 Gy and V12 Gy were the most predictive independent risk factors for radionecrosis (p = 0.0001). The correlation was more significant for symptomatic than asymptomatic brain necrosis. In a subsequent analysis we have evaluated the incidence of events according to the V10 and V12 Gy quarpercentiles distribution. At a median follow-up of 9.4 months V10 Gy radionecrosis rates were 2.6% for volumes <4.5 cm^3 ^(1^st ^quartile, Q1), 11% for volumes of 4.5-7.7 cm^3 ^(2^nd ^quartile, Q2), 24% for volumes of 7.8-12.6 cm^3 ^(3^rd ^quartile, Q3), and 47% for volumes >12.6 cm^3 ^(4^th ^quartile, Q4). The V12 Gy radionecrosis rates were the same for volumes < 3.3 cm^3 ^(Q1), 3.3-5.9 cm^3 ^(Q2), 6.0-10.9 cm^3 ^(Q3), and >10.9 cm^3 ^(Q4). For V10 Gy > 19.1 cm^3 ^and V12 Gy > 15.4 cm^3 ^corresponding to the 90^th ^percentile the risk of radionecrosis was 62%. The actuarial risk at 1 year for the development of brain radionecrosis was 0% in Q1, 16% in Q2, 24% in Q3, and 51% for V12 Gy (Figure 2).

**Table 4:** Univariate and multivariate analysis of radiation-induced brain necrosis

Variables	Univariate analysis	Multivariate analysis
Age	0.2	
Gender	0.3	
KPS	0.04	0.1
Tumor volume	<0.0001	0.02
Dose	0.09	
Conformality index	0.04	0.2
Homogeneity index	0.1	
lung histology	0.1	
Melanoma histology	0.3	
Breast histology	0,2	
frontal location	0.2	
parietal location	0.03	0.1
temporal location	0.3	
cerebellar location	0.2	
occipital location	0.3	
brainstem location	0.9	
V10 Gy	<0.0001	0.001*
V12 Gy	<0.0001	0.001**
V14 Gy	<0.0001	0.005°
V16 Gy	0.0001	0.009°°
V18 Gy	0.001	0.05^

*0.003 for symptomatic and 0.04 for asymptomatic brain necrosis

**0.003 for symptomatic and 0.04 for asymptomatic brain necrosis

°0.01 for symptomatic and 0.1 for asymptomatic brain necrosis

°°0.02 for symptomatic and 0.3 for asymptomatic brain necrosis

^0.03 for symptomatic and 0.5 for asymptomatic brain necrosis

### Salvage treatment for intracranial/local progression

Forty-seven patients received WBRT and 21 patients received further SRS for intracranial progression. Patient receiving WBRT were subsequently excluded from the analysis. Among these patients, the median time to progression was 6 months (range 2-32 months). Median survival after WBRT was 6.7 months. Local progression was treated with resection in 8 patients and WBRT or SRS in 6 patients. Histopathological evaluation of surgically treated lesions showed tumor progression in all patients.

## Discussion

In the present study we have evaluated the clinical outcome and the risk of treatment-related complications in 206 patients treated with SRS as initial treatment for 1-3 brain metastases. Median overall survival and brain control were 14.1 months and 10 months, respectively. The 1-year and 2-year survival rates were 58% and 24%, and respective brain control rates were 43% and 22%. Sixteen patients recurred locally after SRS with 1-year and 2-year local control of 92% and 84%, respectively. The reported results are in accordance with previous series of SRS for brain metastases that report a median survival ranging from 7 to14 months [1-8].

Surgery, WBRT, and SRS alone or in combination have been employed as treatment option for patients with either single or multiple brain metastases, although their optimal treatment is still an issue that remains open for debate. Survival advantages with the use of SRS alone or in conjunction with WBRT have been reported by several randomized trials [2,5,7,8]. In a series of 132 patients with 1-4 brain metastases randomly assigned to receive WBRT plus SRS or SRS alone Aoyama et al [7] reported no significant difference in survival (8 months versus 7.5 months) and 1-year local control (72.5% versus 88.7%). Although SRS alone was associated with increased intracranial progression as compared with WBRT plus SRS, no differences in the frequency of neurologic deaths and preservation of neurologic function were observed. Similarly, the recent EORTC 22952-26001 study on the adjuvant WBRT versus observation after SRS or surgical resection of 1-3 cerebral metastases showed that adjuvant WBRT was able to reduce the frequency of intracranial progression but failed to improve the median survival [8]. Few studies have compared SRS with or without WBRT versus resection plus WBRT, with the majority of them reporting no differences in survival and neurological deaths between groups [31-35]. In a retrospective analysis of 206 patients with one or two metastases, Rades et al [35] reported a similar outcome in patients treated with WBRT plus SRS or surgery plus WBRT and boost. The 1-year survival and brain control rates were 65% and 70% after WBRT plus SRS, and 63% and 78% after surgery plus WBRT and boost, respectively. Based on the present results and published data, SRS alone as initial treatment strategy in patients with either single or multiple metastases is a feasible therapeutic option associated with high local control and survival benefits, although the superiority of SRS versus other treatment options in terms of improved survival remains to be demonstrated. Certainly, the high 1-year brain tumor recurrence rates of about 50% after SRS alone clearly indicates that a frequent monitoring of intracranial disease is mandatory for such patients.

On multivariate analysis, KPS >70 and stable extracranial disease were significantly associated with longer survival. Number of metastases did not emerge as significant variable associated with the outcome similarly to some recent [5,6,17] and differently from earlier published series [12,36]. The high local control after SRS and the improved control of extracranial disease reported with the combination of cytotoxic and targeted agents [37-41] may, at least in part, explain these results. Similarly, older age did not have a negative impact on survival, suggesting that SRS is a feasible and safe approach also in this subgroup of patients [42,43].

Brain necrosis represents the most important late toxicity reported after SRS, leading to neurological complications in 2-32% of patients [1-10,18-20]. At doses of 16-22 Gy usually employed for the radiosurgical treatment of brain metastases, radionecrosis has been reported in up to 50% of treated lesions, with radiation dose, tumor volume and location of the lesion being the most important predictive variables [22-26]. In our study, radionecrosis occurred in 24% of treated lesions with SRS, leading to severe neurological complications (RTOG Grade ≥ 3) in 5.8% of patients. Other adverse events included headache, iatrogenic Cushing syndrome, and more rarely conditions as haemorrhage and hydrocephalus. The present results confirm that SRS is associated with a relatively high rate of treatment-related complications as reported by some authors, although with an acceptable incidence of severe neurological deficits [18-21].

Analysis of risk factors for brain necrosis showed that V10 Gy and V12 Gy were the most important independent predictors of both symptomatic and asymptomatic radionecrosis. At a median follow-up of 9.4 months the actuarial risk at 1 year for the development brain radionecrosis increased significantly through Q1-Q4, being 0% in Q1, 16% in Q2, 24% in Q3, and 51% in Q4. Our data are consistent with previous studies that have shown a significant correlation between volume receiving a dose of 10 or 12 Gy and the development of radionecrosis in patients treated with SRS for brain metastases and other intracranial tumors [21,22,25,26]. Blonigen et al [26] in a series of 63 patients with a total of 173 brain metastases treated with SRS have reported a significant radionecrosis risk up to 68.8% for V10 Gy >14.5 cm^3 ^and V12 Gy >10.8 cm^3^, respectively. In contrast, no cases of radionecrosis were found for V10 Gy < 0.68 cm^3 ^and V12 Gy < 0.5 cm^3^. In a retrospective analysis of 198 intracranial tumors treated with Gamma Knife SRS, Korytko et al [25] confirmed the correlation between the V12 Gy and the risk of symptomatic radionecrosis. The risk was 55.3% for V12 Gy > 10 cm^3 ^versus 22.5% for V12 Gy < 10 cm^3^, being significant in multivariate analysis. In contrast, the risk for asymptomatic radionecrosis did not increase with V12 Gy, remaining at 19.1% for tumors <10 cm^3 ^and 18.5% for tumors > 10 cm^3^, respectively. Few authors have evaluated the predictive value of volume receiving 10 or 12 Gy on the development of radionecrosis after SRS for arteriovenous malformation (AVM) [21,22]. At a median follow-up of 28 months Voges et al [22] reported an actuarial risk of radionecrosis of 38.4% at 2 years in 62 patients with intraparenchymal lesions, with an incidence of events of 0% for volumes covered by the 10 Gy isodose-line ≤10 cm^3 ^and 23.7% for volumes >10 cm^3^. Flickinger et al [21] in a series of 307 patients with AVM who received GK SRS at the University of Pittsburgh between 1987 and 1993 observed an incidence of symptomatic radionecrosis of 30.5% at 7 years. On multivariate analysis, V12 Gy and AVM location were the only independent variable that correlated significantly with brain necrosis.

Although the reported risk of radionecrosis after SRS is variable in the published series depending on different radiosurgical techniques, type of lesion treated, length of follow-up and patient's selection, nevertheless volume receiving 12 Gy may be adopted as the standard method of reporting the dose to the normal brain to estimate the risk of toxicity after SRS. In our department brain metastases with a V12 Gy >8.5 cm^3^, which is the midpoint of 3^rd ^quartile corresponding to the risk of developing radionecrosis >10% at 1 year, are considered for hypofractionated stereotactic radiotherapy using a dose of 24-27 Gy in 3 fractions rather than single SRS to reduce the risk of treatment-related complications.

In conclusion, SRS represents a feasible option for patients with brain metastases associated with survival benefit, however a significant subset of patients may develop neurological complications. Radionecrosis represents the most important late toxicity after SRS with the brain volumes irradiated at 10 and 12 Gy being the most important independent predictors of brain necrosis. Large lesions at high risk of radiation-induced complications especially when located in/near eloquent areas should be considered for hypofractionated stereotactic radiotherapy.

## Competing interests

The authors declare that they have no competing interests.

## Authors' contributions

GM conceived the study, participated in its design and coordination, and drafted the manuscript. GL, GT and AR participated in study design, analysis and interpretation of data, and helped to draft the manuscript. EC and MFO performed the statistical analysis and participated in acquisition and analysis of data. AB and RME critically reviewed/revised the article. All authors read and approved the final manuscript.
